# PD‐1 blockade enhances the effect of targeted chemotherapy on locally advanced pMMR/MSS colorectal cancer

**DOI:** 10.1002/cam4.7224

**Published:** 2024-06-18

**Authors:** Fengyun Pei, Wan He, Yinghua Duan, Qijun Yao, Yandong Zhao, Xinjuan Fan, Shuai Liu, Haiyang Chen, Fang He, Tingzhi Liu, Jiaoting Chen, Yijia Zheng, Heping Li, Xiaofang Guo, Lishuo Shi, Li Ling, Yaoxu Chen, Jiapeng He, Miao Liu, Mengli Huang, Yuezong Bai, Jianping Wang, Meijin Huang, Jun Huang

**Affiliations:** ^1^ Department of Colorectal Surgery, The Sixth Affiliated Hospital Sun Yat‐sen University Guangzhou China; ^2^ Department of General Surgery, The Sixth Affiliated Hospital Sun Yat‐sen University Guangzhou China; ^3^ Department of Oncology Shenzhen People's Hospital (The Second Clinical Medical College, Jinan University, The First Affiliated Hospital, Southern University of Science and Technology) Shenzhen China; ^4^ Department of Traditional Chinese Medicine, The First Affiliated Hospital Sun Yat‐sen University Guangzhou China; ^5^ Department of Pathology, The Sixth Affiliated Hospital Sun Yat‐sen University Guangzhou China; ^6^ Department of Radiation Oncology, The Sixth Affiliated Hospital Sun Yat‐sen University Guangzhou China; ^7^ Department of Hematology, The Sixth Affiliated Hospital Sun Yat‐sen University Guangzhou China; ^8^ Department of Medical Oncology of the Eastern Hospital, The First Affiliated Hospital Sun Yat‐Sen University Guangzhou China; ^9^ Clinical Research Center, The Sixth Affiliated Hospital Sun Yat‐sen University Guangzhou China; ^10^ Faculty of Medical Statistics, School of Public Health Sun Yat‐sen University Guangzhou China; ^11^ Medical Affairs 3D Medicines, Inc. Shanghai China; ^12^ Guangdong Provincial Key Laboratory of Colorectal and Pelvic Floor Diseases, The Sixth Affiliated Hospital Sun Yat‐sen University Guangzhou China; ^13^ Guangdong Institute of Gastroenterology Guangzhou China

**Keywords:** colorectal cancer, neoadjuvant treatment, PD‐1 blockade, pMMR/MSS

## Abstract

**Background:**

Patients with DNA mismatch repair‐proficient/microsatellite stable (pMMR/MSS) colorectal cancer (CRC), which accounts for 85% of all CRC cases, display a poor respond to immune checkpoint inhibitors (i.e., anti‐PD‐1 antibodies). pMMR/MSS CRC patients with locally advanced cancers need effective combined therapies.

**Methods:**

In this pilot study, we administered six preoperative doses of each 2‐week cycle of the anti‐PD‐1 antibody sintilimab (at a fixed dose of 200 mg), oxaliplatin, and 5‐FU/CF (mFOLFOX6) combined with five doses of bevacizumab (the number of doses was reduced to prevent surgical delays) to patients with cT4NxM0 colon or upper rectal cancers. And radical surgery was performed approximately 2 weeks after the last dose of neoadjuvant therapy. The primary endpoint was a pathologic complete response (pCR). We also evaluated major pathologic response (MPR, ≤10% residual viable tumor), radiological and pathological regression, safety, and tumor mutation burden (TMB), and tumor microenvironment (TME) characteristics.

**Results:**

By the cutoff date (September 2023), 22 patients with cT4NxM0 pMMR/MSS colon or upper rectal cancers were enrolled and the median follow‐up was 24.7 months (IQR: 21.1–26.1). All patients underwent R0 surgical resection without treatment‐related surgical delays. pCR occurred in 12 of 22 resected tumors (54.5%) and MPR occurred in 18 of 22 (81.8%) patients. At the cutoff date, all patients were alive, and 21/22 were recurrence‐free. Treatment‐related adverse events of grade 3 or higher occurred in of 2/22 (9.1%) patients. Among the pCR tumors, two were found to harbor POLE mutations. The degree of pathological regression was significantly greater than that of radiological regression (*p* = 1.35 × 10^−8^). The number of CD3+/CD4+ cells in the tumor and stroma in pretreated biopsied tissues was markedly lower in pCR tumors than in non‐pCR tumors (*p* = 0.038 and *p* = 0.015, respectively).

**Conclusions:**

Neoadjuvant sintilimab combined with bevacizumab and mFOLFOX6 was associated with few side effects, did not delay surgery, and led to pCR and non‐pCR in 54.5% and 81.8% of the cases, respectively. Downregulation of CD3/CD4 expression in the tumor and stroma is related to pCR. However, the molecular mechanisms underlying PD‐1 blockade‐enhanced targeted chemotherapy require further investigation.

## 1 INTRODUCTION

Colorectal cancer (CRC) is ranked third in terms of incidence and fourth in terms of mortality worldwide. Approximately 10%–20% of these patients present with T4 colon cancer.^1^ Patients with T4 CRC have poor disease‐free survival (DFS) and overall survival (OS) because R0 resection is difficult to achieve and has a high risk of local failure, especially with regard to peritoneal recurrence.^2^, ^3^ A study showed that patients with T4 CRC had a 5‐year DFS rate of 46.9%, which was markedly lower than that of patients with T1–T3 tumors (T1: 88.4%; T2: 78.2%; T3: 64.8%).^4^ Neoadjuvant treatments may be a potential strategy for shrinking tumors and reducing the risk of incomplete excision and micrometastasis.

Importantly, the FOxTROT phase III clinical trial is the first to investigate the pathological response of locally advanced colon cancer to neoadjuvant treatment.^5^ Six‐week treatment with mFOLFOX6 or XELOX as neoadjuvant chemotherapy for resectable colon cancer was delivered safely with lower perioperative morbidity and significant pathological downstaging, including a pCR rate of 3.3%. This study also observed a trend towards improved disease control at 2 years of age. Additionally, T4 colon cancer seemed to benefit more from neoadjuvant chemotherapy (mFOLFOX6) (HR = 0.65) than T3 colon cancer.^6^ Neoadjuvant radiotherapy may also be considered for selective patients with T4 colon cancer invading a fixed structure, with reported pCR rates of 4%–38.1%.^7^ However, radiotherapy may enhance the surgical difficulties and induce colitis, perforation, and bone marrow suppression. Additionally, it remains unclear whether the downstaging effect of neoadjuvant radiotherapy in patients with clinical T4 colon cancer can translate to survival benefits. Thus, further phase III randomized trials and the exploration of new neoadjuvant regimens with higher efficacy and tolerability are warranted.

Neoadjuvant immunotherapy is attractive because early stage cancers may be more responsive to anti‐PD‐1 antibodies owing to a lower proportion of immunosuppressed hosts and tumor‐intrinsic factors.^8^ The NICOLE study reported in ESMO 2020 showed that 27.8% (5/18) of patients with DNA mismatch repair‐proficient/microsatellite stable (pMMR/MSS) colon cancer achieved a complete response (CR) or partial response (PR),^9^ which is similar to the data on pMMR colon cancer (27%) in the NICHE study.^10^ Bevacizumab, a VEGF monoclonal antibody, is often used synergistically with anti‐PD‐1 antibodies for its immune‐supportive effects.^11^

Immune checkpoint inhibitors (ICIs) have been confirmed to significantly improve the efficacy of dMMR/MSI‐H metastatic CRC and recommended as the first‐line treatment.^12^, ^13^ However, single‐agent ICIs have been shown to be ineffective for patients with pMMR/MSS CRC. The safety and efficacy of chemotherapy combined with AADs and ICIs was verified in pMMR/MSS mCRC,^14^, ^15^ it's activity in pMMR/MSS locally advanced CRC remains unclear. pCR was observed only in 4%–7% locally advanced CRC patients who received conventional neoadjuvant chemotherapy in previous study.^5^, ^16^ Therefore, neoadjuvant therapy of mFOLFOX6 with bevacizumab and PD‐1 blockade may be a potential treatment strategy to better achieve tumor regression and increase R0 resection rates for pMMR/MSS locally advanced CRC patients. Moreover, no study has explored the immune microenvironment of sintilimab in combination with mFOLFOX and bevacizumab in patients with pMMR/MSS locally advanced CRC.

In this study, the neoadjuvant therapy regimen of sintilimab combined with mFOLFOX6 and bevacizumab was evaluated for efficacy and safety in cT4NxM0 pMMR/MSS colon or upper rectal cancer with a primary endpoint of pathologic complete response (pCR).

## 2 METHODS

### 2.1 Patients

Using a descriptive case series study approach, this study retrospectively analyzed 22 patients (15 males and 7 females, aged from 37 to 79 years old) cT4NxM0 pMMR/MSS colon or upper rectal cancer who were treated in the Sixth Affiliated Hospital of Sun Yat‐sen University from December 2020 and March 2022. All patients had an Eastern Cooperative Oncology Group performance status score of 0 or 1 (on a 5‐point scale, in which higher numbers represent greater disability), normal organ function, and sufficient pulmonary function.^17^ Key exclusion criteria were ongoing systemic immunosuppressive therapy, immunodeficiency, presence of infectious or active autoimmune diseases, and clinically significant concurrent cancer.

### 2.2 Study design

This retrospective exploratory single‐group study was developed by the authors and performed at the Sixth Affiliated Hospital of Sun Yat‐Sen University in China. The patients received six doses of intravenous sintilimab (at a fixed dose of 200 mg), mFOLFOX6 (oxaliplatin at a dose of 85 mg/kg of body weight [mg/m^2^], CF at a dose of 400 mg/m^2^, a bonus injection of 5‐FU at a dose of 400 mg/m^2^ and a continuous injection of 5‐FU [at a dose of 2400 mg/m^2^ for 48 h]) plus bevacizumab (at a dose of 5 mg/kg) every 2 weeks. Radical surgery was conducted approximately 2 weeks after the last dose of neoadjuvant therapy without bevacizumab. The workflow of the study design is shown in Figure S1. Written informed consent was obtained from all patients.

Adverse events in all patients were categorized according to the National Cancer Institute Common Terminology Criteria for Adverse Events version 5.0.^18^ Feasibility was defined as any delay in the planned surgery of no more than 28 days (i.e., a surgical delay of >21 days and 7 days of scheduling). All patients underwent baseline tumor staging, including pathological evaluation of the primary tumors using colonoscopy, contrast‐enhanced computed tomography (CT) of the chest and abdomen, and magnetic resonance imaging (MRI) of the brain and abdomen. CT or MRI scans were repeated within 7 days before surgery. Changes in tumor size were assessed according to the Response Evaluation Criteria in Solid Tumors (RECIST), version 1.1.^19^ Resection of the primary tumor and lymph nodes was performed according to institutional standards. None of the patients who achieved pCR received adjuvant therapy, while those who did not receive pCR were offered the same neoadjuvant therapy regimen of sintilimab combined with mFOLFOX6 and bevacizumab for 4–6 cycles; upon the clinical completion of this regimen, they were followed to determine DFS and OS.

To identify potential biomarkers related to neoadjuvant therapy response, pretreated biopsied tissues from 22 patients all underwent DNA sequencing. The process of cancer initiation and progression is considered to be constant, dynamic, and reciprocal interactions between cancer cells and the tumor microenvironment (TME). To determine the relationship between TME and different therapeutic responses, multiplex immunofluorescence (mIF) was performed on samples from 20 patients without POLE mutations.^20^ We also assessed CD3, CD4, CD8, CD20, and PD‐1 expression via immunohistochemical staining in the preoperative and postoperative tissues of the four patients (Figure S2).

### 2.3 Pathological assessments

Primary colorectal tumors and lymph node surgical specimens were staged according to the criteria of the American Joint Committee on Cancer (8th edition) to evaluate tumor invasion depth and affected lymph nodes.^21^ The percentage of residual viable tumor was identified with routine hematoxylin and eosin staining for primary tumor assessment, and a major pathologic response was considered if the tumors had no more than 10% viable tumor cells.^22^ The immunohistochemical results are presented in the Methods section of the appendix—Data S1.

### 2.4 Immunohistochemical staining and DNA sequencing

The results of immunohistochemical and next‐generation sequencing analyses of tumors are described in the Methods section of the Appendix—Data S1. Briefly, we compared various mutations in the DNA of pretreated tumors from patients with and non‐pCR to identify the genes associated with treatment responses.

### 2.5 mIF investigation of the TME

All non‐cancerous host cells in the tumor, including adaptive and innate immune cells, fibroblasts, endothelial cells, neurons, and their noncellular components, including the extracellular matrix (ECM) and soluble products such as chemokines, cytokines, growth factors, and extracellular vesicles are recognized to make up TME.^20^ Details regarding the TME, which was examined with pretreatment of tumor samples and matched normal tissue samples, and bioinformatic analyses are provided in the Methods section of the Appendix—Data S1. By comparing the tumor and its adjacent stroma, potential biomarkers of good response can be identified.

### 2.6 Statistical analysis

To analyze the tumor specimens, including the pathological, genomic, and immunofluorescent data, T tests and Wilcoxon tests were performed to explore the differences between the two groups. The Kruskal–Wallis test was performed to explore the differences among the three groups. Pearson's correlation analysis was performed to identify associations between the two groups. The reported *p*‐values were two‐tailed, and a *p* value <0.05 was set as statistical significance, no power analysis was performed in this exploratory cohort. Kaplan–Meier curves with log‐rank tests were used to calculate overall cumulative probabilities. All analyses in this study were conducted using R4.1.2 for windows; figures were generated using the same software.

## 3 RESULTS

### 3.1 Characteristics of the patients

The clinical characteristics of the enrolled patients are summarized in Table 1. All 22 patients with pMMR/MSS CRC received six doses of sintilimab plus mFOLFOX6 combined with bevacizumab (without a sixth cycle to prevent surgical delays). Among these patients, 22/22 had adenocarcinoma, 9/22 had cT4N+M0 upper rectal cancer and 13/22 had cT4N+M0 colon cancer.

**TABLE 1:** Characteristics of the patients at baseline, according to pathologic response.

Characteristics	All patients (*N* = 22)	Patients with complete pathologic response (*N* = 12)	Patients with noncomplete pathologic response (*N* = 10)	*p* value
Age, years				
Mean ± SD	61.2 ± 12	59.9 ± 14.4	62.8 ± 8.7	0.59
Median (range)	65 (37–79)	59 (37–79)	66 (45–74)	
Sex, no. (%)				
Female	7 (31.8)	4 (33.3)	3 (30)	0.88
Male	15 (68.2)	8 (66.7)	7 (70)	
Tumor (%)				
T4a	14 (63.6)	6 (50)	8 (80)	0.16
T4b	8 (36.4)	6 (50)	2 (20)	
Lymph node (%)				
N1	6 (27.2)	4 (33.3)	2 (20)	0.51
N2	16 (72.7)	8 (66.7)	8 (80)	
Site				
Upper rectal	9 (41)	4 (33.3)	5 (50)	0.45
Colon	13 (59)	8 (66.7)	5 (50)	
Histologic type				
Adenocarcinoma	22 (100)	12 (100)	10 (100)	0.9
Well differentiated	6 (27.2)	4 (33.3)	2 (20)	
Moderately differentiated	14 (63.6)	6 (50)	8 (80)	
Poorly differentiated	2 (9.1)	2 (16.7)	0	

### 3.2 Clinical activity

**FIGURE 1:**
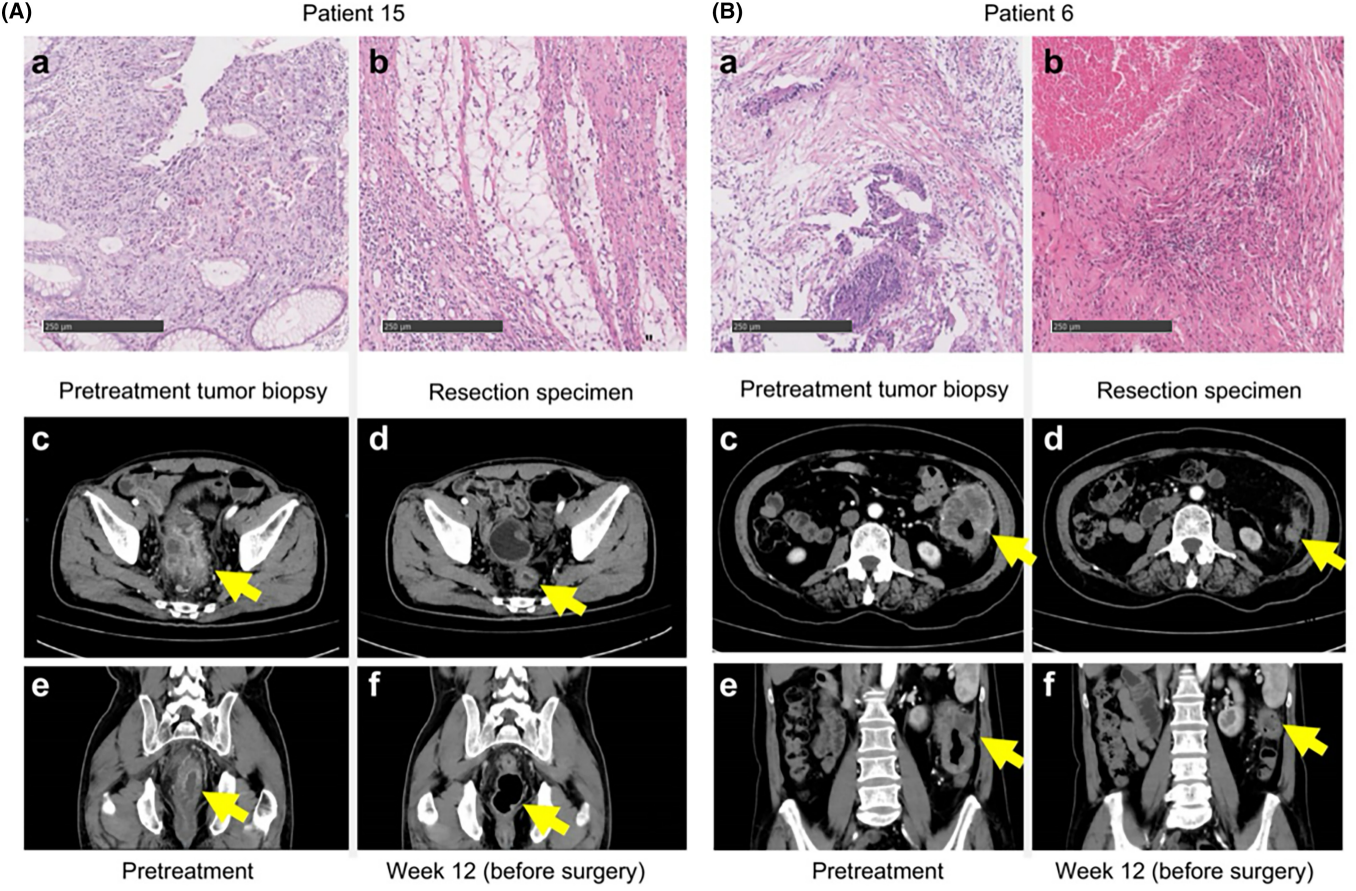
Patterns of pathologic and radiologic responses to neoadjuvant therapy with PD‐1 antibody in combination with mFOLFOX6 and bevacizumab. (A) The upper row shows representative sections of tumor specimens obtained from an adult patient A with cT4bN2M0 middle rectal cancer before (a) and after (b) administration of the cocktail regimen (hematoxylin and eosin staining). The patient showed 100% pathological regression of the tumor in the rejected specimen. The lower row shows computed tomography (CT) scans of the patient's abdomen before (c, e) and 12 weeks after (d, f) administration of the cocktail regimen. A scan performed before surgery showed 78% of shrinkage. (B) The upper row shows representative sections of tumor specimens obtained from another adult patient B with cT4aN1bM0 sigmoid colon cancer before (a) and after (b) administration of the cocktail regimen (hematoxylin and eosin staining). The patient showed 100% pathological regression of the tumor in the resected specimen. The lower row shows CT scans of the patient's abdomen before (c, e) and 12 weeks after (d, f) administration of the cocktail regimen. A preoperative scan performed before surgery showed 79% shrinkage.

**FIGURE 2:**
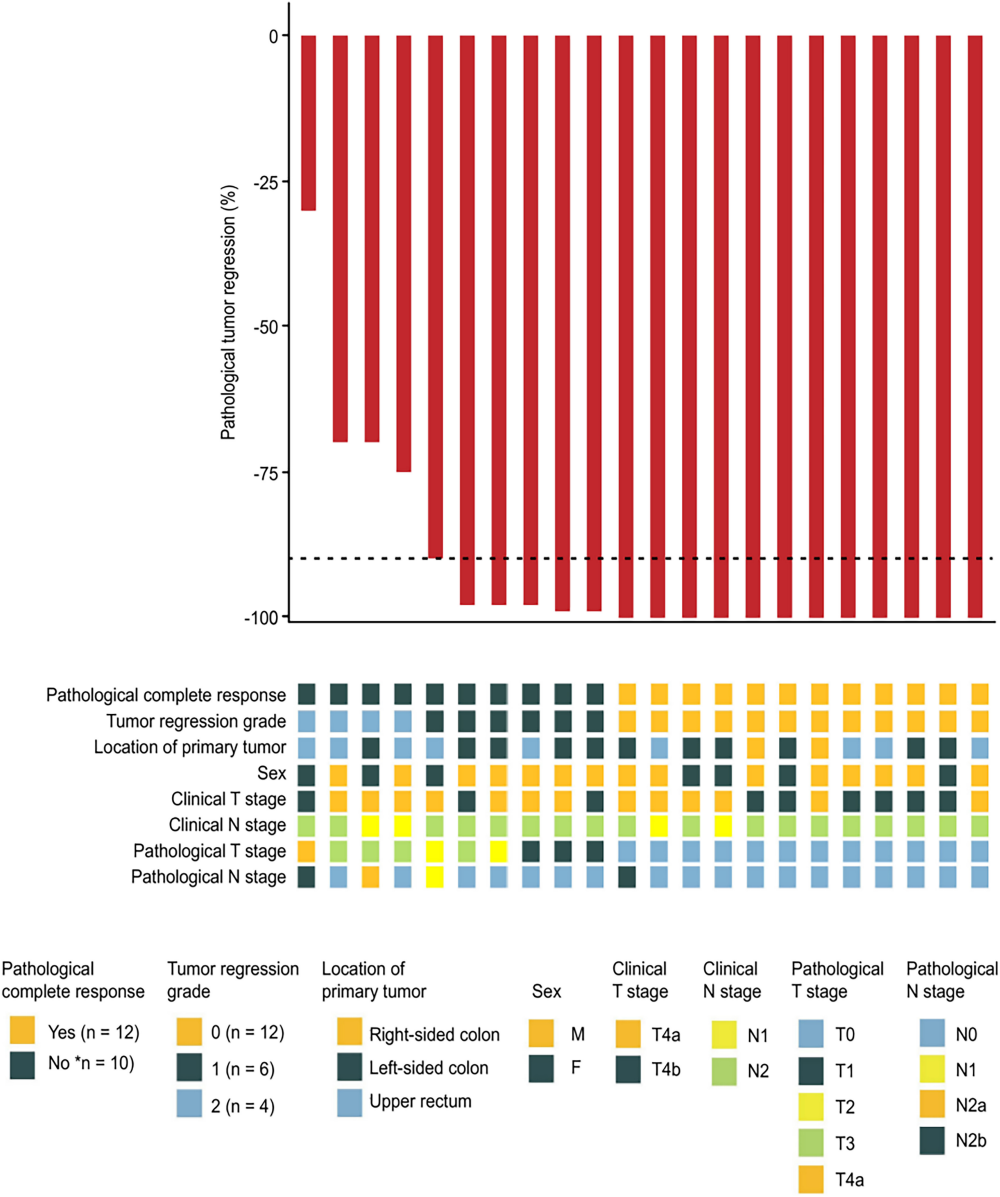
Pathologic assessment of response to neoadjuvant therapy with a PD‐1 antibody in combination with mFOLFOX6 and bevacizumab. Pathological regression in cT4a/4b locally advanced colorectal cancer (CRC) after neoadjuvant administration, according to the percentage of remaining viable tumor cells, for each of the 22 patients who underwent surgical resection. The black dotted line indicated the threshold for a major pathological response (MPR), indicating 90% regression. Clinical features, including tumor regression grade (National Comprehensive Cancer Network criteria), tumor location, sex, radiological staging, and T and N staging of the surgical specimen, were displayed for each patient.

**FIGURE 3:**
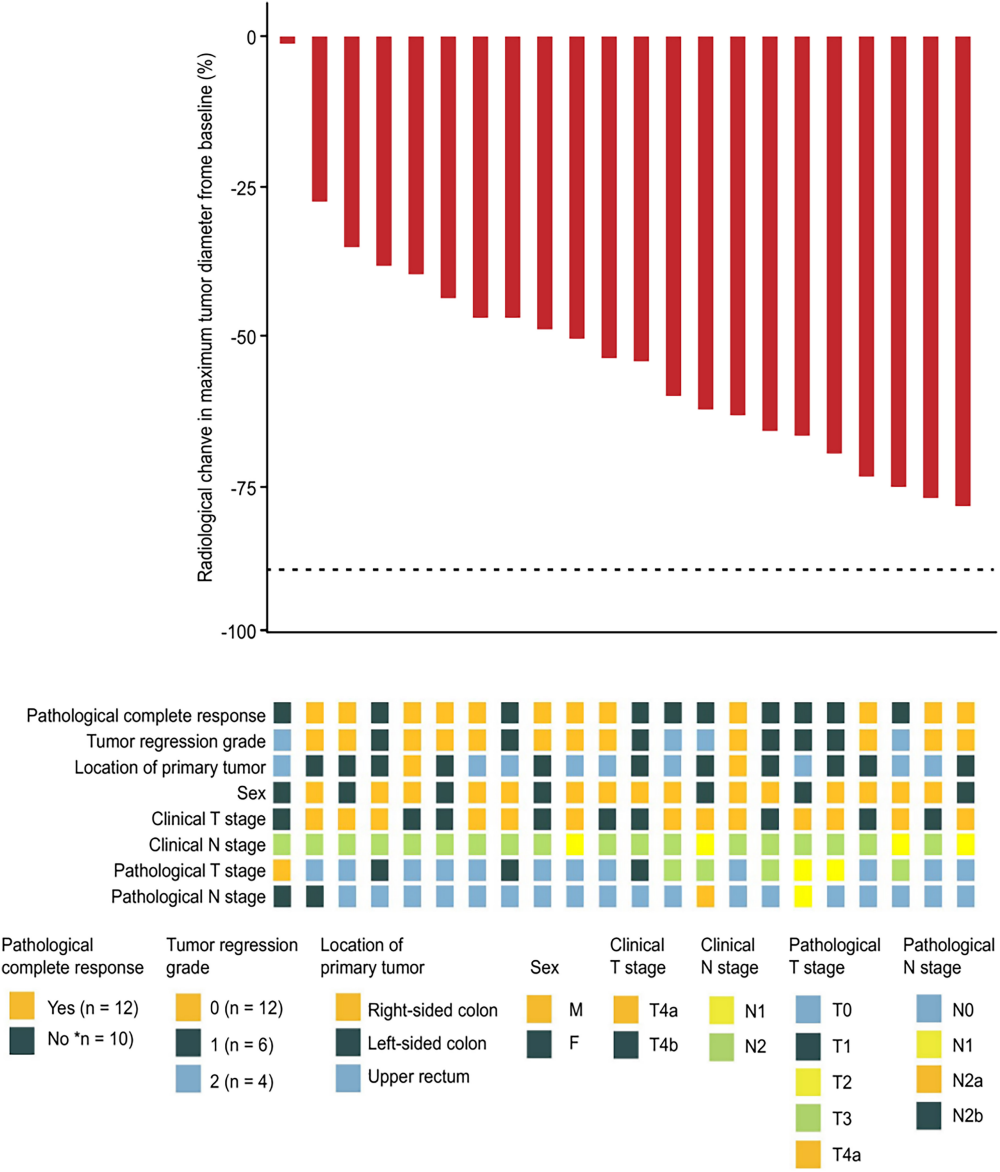
Radiological assessment of response to neoadjuvant therapy with a PD‐1 antibody in combination with mFOLFOX6 and bevacizumab. Pathological regression of the resected primary colorectal cancer (CRC) tumor after neoadjuvant administration of PD‐ 1 antibody plus mFOLFOX6 and bevacizumab, according to the percentage of remaining viable tumor cells for each of the 22 patients who underwent surgical resection. The black dotted line indicates the threshold for major pathological response (90% regression). Clinical features, including tumor regression grade (National Comprehensive Cancer Network criteria), tumor location, sex, radiological staging, and T and N staging of the surgical specimen, were displayed for each patient.

Representative radiologic and pathologic responses after six preoperative doses of sintilimab combined with mFOLFOX6 plus five doses of bevacizumab are shown in Figure 1. Among the 22 patients who underwent resection, pathological down staging from the pretreatment clinical stage occurred in 20 (90.9%) (Figure 2; Table S1). Of the patients who had evaluable radiographic results, none exhibited CR, 20 (90.9%) exhibited PR, and 2 (9.1%) had stable disease (SD) (Figure 3; Table S1). Notably, the pathologic response was significantly greater than the radiologic response (*p* = 1.35 × 10^−8^, Figure S3). As of the cutoff date (September 2023), at a median of 24.7 months of postoperative follow‐up (range: 15.0–29.7), 21 of 22 patients (95.5%) who underwent surgical resection were alive, and 21 of 22 (95.5%) were recurrence‐free (Figure S4).

### 3.3 Genomic analyses

To investigate the influence of genomic alterations, TMB, and their potential correlations with pathological response, we conducted DNA sequencing of pretreatment tumor biopsies from 22 patients with adequate available tissue (Figure S5). Among the pMMR/MSS CRC patients without POLE mutations, a median of three somatic mutations (range: 0–8) per tumor were detected. All resected tumors from the 22 patients who provided samples for sequencing were evaluated for tumor response. There was no correlation between pathological response and pathogenic mutations, a finding similar to that of the mutation burden (Figures S6 and S7).

### 3.4 mIF investigation of the TME

**FIGURE 4:**
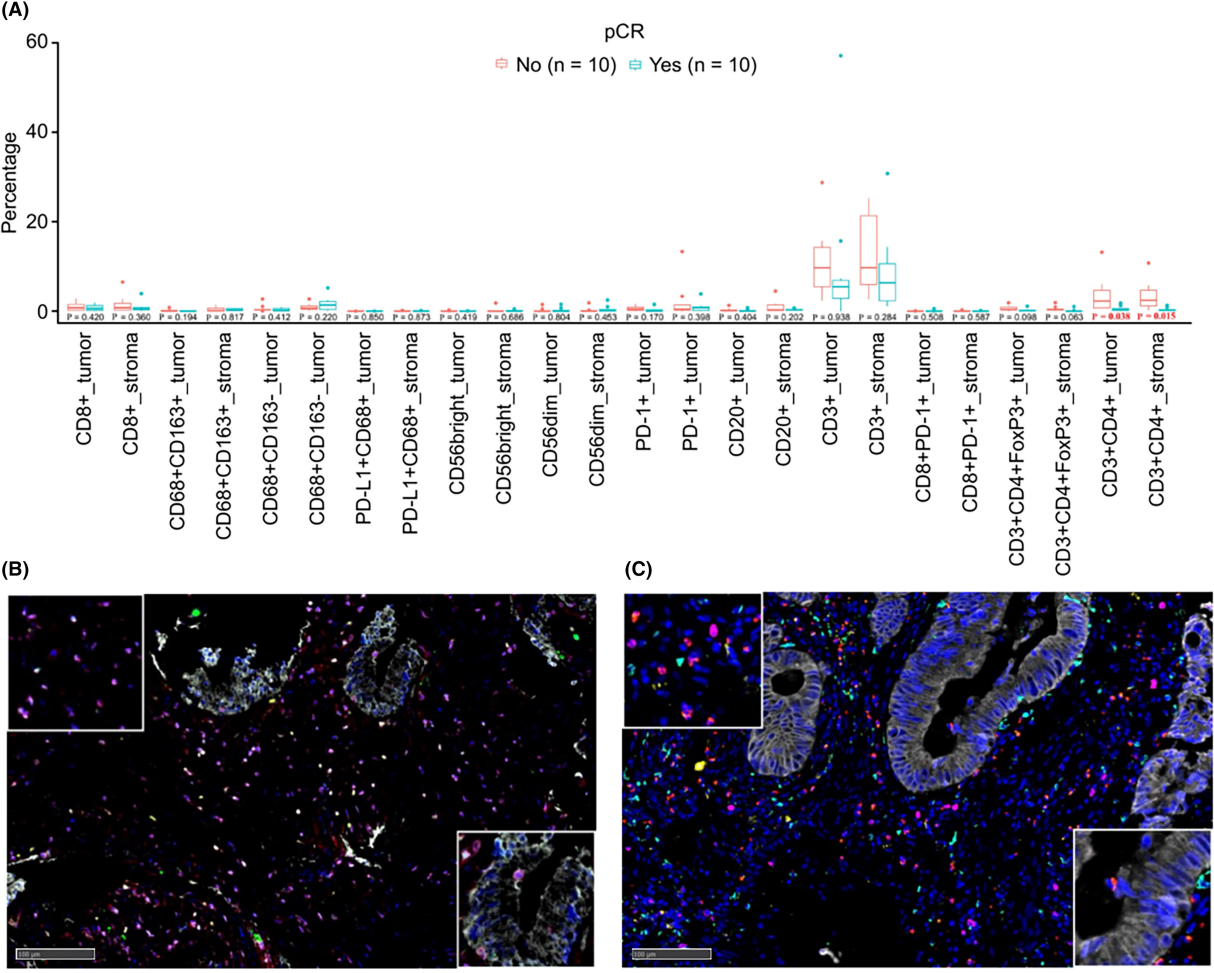
Correlation between TME characteristics before neoadjuvant treatment and tumor regression grade. Correlations between tumor microenvironment (TME) characteristics and pathological responses are shown. (A) TME characteristics of pCR and non‐pCR patients. Patients with non‐pCR ahd a higher CD3+CD4+ percentage than those with pCR in the tumor (*p* = 0.038; Wilcoxon test) and stromal regions (*p* = 0.015 Wilcoxon test). Representative images of pCR patient no. 14 (B) and non‐pCR patient no. 2 (C). Multiplex immunofluorescence (mIF) staining was performed using an Akoya OPAL Polaris 7‐Color Automation IHC Kit (NEL871001KT). FFPE tissue slights were incubated with primary antibodies against CD3 (pink; Dako, A0452), CD4 (red; Abcam, ab133616, 1:100), CD56 (cyan: Abcam, ab75813, 1:100) to identify different immune cell subtypes (CD3+ T‐cells in the stromal region. The lower right boxes represent CD3+CD4+ T cells in the tumor region.

We conducted mIF on samples from 20 patients without POLE mutations to obtain a glimpse of the TME. The percentages of different immune cell subtypes, including CD3+ T cells, CD4+ T cells, CD8+ T cells, regulatory T cells, macrophages, NK cells, and B cells, in the tumor and stromal regions were quantified. Patients with non‐pCR had significantly more CD3+CD4+ T cells than those with pCR in the tumoral and stromal regions (Figure 4; *p* = 0.038 and *p* = 0.015, respectively). No differences were observed in the percentages of other immune cell subtypes between the pCR and non‐pCR groups. TME of different tumor regression grades (TRG scores) was also explored. Patients with TRG1 expression had a higher percentage of CD3+CD4+ cells in the stromal region than those with TRG0 or TRG2 expression (Figure S8, *p* = 0.020).

### 3.5 Correlation between the neutrophil‐to‐lymphocyte ratio at diagnosis and before surgery and the percentage of residual tumor

The neutrophil‐to‐lymphocyte (NLR) of each of the 22 patients was determined at two time points: at diagnosis and before surgery. There was no correlation between NLR and the percentage of residual tumors (Pearson correlation = 0.35, *R* ^2^ = 0.1235). Additionally, the number of pathogenic mutations did not correlate with radiological tumor regression (Pearson correlation = 0.0048, *R* ^2^ = 2 × 10^−5^) (Figure S9).

### 3.6 Safety and feasibility

Neoadjuvant treatment was not associated with any of the previously reported toxic effects. Treatment‐related adverse events occurred in 21 of the 22 patients (95.5%), but only two events were grade 3 or higher (Table S2). None of the patients experienced treatment‐related surgical delays (according to the protocol definition). The median interval between administration of the sixth dose of sintilimab and surgery was 19 days (range, 12–50 days), and all eligible patients underwent R0 tumor resection. No major surgery‐related complications (≥grade 3) occurred according to the Clavien–Dindo classification.

## 4 DISCUSSION

Our neoadjuvant sintilimab combination regimen in patients with cT4NxM0 pMMR/MSS CRC was associated with fewer severe adverse events and no delay in planned surgery, leading to a pCR rate of 54.5% (12/22 patients) and an MPR rate of 81.8% (18/22 patients) in resected tumors, including in two POLE‐mutated patients. Despite the high rates of pCR and MPR on histological examination, none of the patients achieved CR radiologically. The objective response rate (ORR) was 90.9%, which was much higher than the 60% reported in the Checkmate 9X8 trial, in which the group with metastatic CRC received first‐line nivolumab combined with mFOLFOX6 and bevacizumab.^23^ This difference is consistent with tumors being more responsive to anti‐PD‐1 antibodies in early stage CRC because of a lower level of immunosuppressed hosts and tumor‐intrinsic factors.^8^, ^24^

We also found that the pathologic responses were superior to the radiologic responses in pMMR/MSS CRC patients after the combined therapy, similar to the response to PD‐1 blockade in dMMR/MSI‐H CRC patients.^24^ Twelve patients in whom tumors exhibited 29%–79% shrinkage in size on pre‐surgical CT scans were found to have no residual tumors in the surgical specimen. All 18 patients whose tumors had an MPR exhibited less than 80% shrinkage in size on pre‐surgical CT scans. The median radiological regression rate was 52% in the patients with pCR. We assumed that this process occurred because of the residual stroma in the tumor after tumor necrosis rather than the true residual tumor cells. We further evaluated the pathological changes in the pMMR/MSS CRC tumors. Interestingly, we found that immune cell infiltration was more evident in the residual tumors than in the pretreatment biopsies. The increase in immune cell infiltration in residual tumors may be associated with PD‐1 blockade.^25^ In contrast, little immune cell infiltration was observed in the stroma of pCR specimens. We assumed that this difference was due to the evacuation of immune cells after the complete clearance of the tumor cells.

However, in contrast to PD‐1 blockade in patients with dMMR/MSI‐H CRC, the achievement of pCR and MPR in patients with pMMR/MSS CRC was not associated with increased TMB or pathogenic mutations (Figure S7). Studies have shown that chemotherapy and bevacizumab may improve the TME.^26^, ^27^ Therefore, in the present study, we hypothesized that the enhanced efficacy of PD‐1 blockade treatment is related to changes in TME caused by targeted chemotherapy. However, because few immune cells were found in pCR specimens, it was difficult to determine the exact TME changes between pCR and non‐pCR specimens. Additionally, we evaluated NLR, which has been reported to be associated with the immune response to PD‐1 blockade,^28^ patients at diagnosis and before surgery. However, increased NLR was not related to the percentage of residual tumors.

Our study is the first to report that the downregulated expression of CD3+ and CD4+ T cells may be correlated with pCR and tumor regression induced by administration of a PD‐1 antibody in pMMR/MSS CRC patients. In this study, the expression of CD3+ and CD4+ cells were altered before and after treatment. In patients without pCR, CD3+ and CD4+ T cell infiltration increased after treatment. In contrast, these cells were barely observed in the tumoral regions of the patients with pCR after treatment. This result suggests that immune cells had already been retreated in pCR patients but aggregated in non‐pCR patients. This is consistent with the results of a study by Chalabi et al., which showed that immune infiltration in non‐pCR patients increased after treatment.^10^ As demonstrated for the first time in this study, the percentage of CD3+CD4+ T cells in pretreatment biopsies may serve to predict immunotherapy response in pMMR/MSS CRC patients. Similarly, Yasuda et al. demonstrated that lymphocyte infiltration before neoadjuvant chemotherapy in rectal cancer biopsy samples was closely associated with tumor regression.^29^ Additionally, Jary et al. divided immune infiltration into the invasive front and intratumoral regions and found that the expression of CD3 in the invasive front was higher in patients with higher TRG scores,^30^ which is similar to the trend observed in our study, but contrary to the findings of Mlecnik et al.^31^ Liu et al. collected samples during neoadjuvant therapy and found that the lowest absolute lymphocyte count was significantly higher in patients who achieved a pathologic response.^32^ However, we did not conduct sampling during neoadjuvant therapy in the present study.

The limitations of our study include, but are not limited to, the small sample size and the short postoperative follow‐up. Larger studies and long‐term follow‐up are needed to confirm the most predictive biomarkers of patient response to neoadjuvant PD‐1 blockade combined with targeted chemotherapy for treating patients with locally advanced pMMR/MSS CRC, and to examine the associations between the pathologic response resulting from this combined strategy and disease‐free and OS. Based on this pilot study, we registered a phase II clinical trial that is currently recruiting participants to further investigate the effect of this neoadjuvant PD‐1 combination regimen on the treatment of locally advanced (cT4NxM0) pMMR/MSS CRC (ClinicalTrials.gov, NCT04895137).

## 5 CONCLUSION

Neoadjuvant sintilimab combined with mFOLFOX6 and bevacizumab for locally advanced pMMR/MSS CRC was associated with fewer side effects, did not delay surgery, and led to pCR and MPR rates of 54.5% and 81.8%, respectively. Downregulation of CD3/CD4 T cell infiltration in pretreatment tumors may be associated with a higher possibility of pCR. The combined PD‐1 blockade regimen for treating locally advanced pMMR/MSS CRC merits further investigation.

## AUTHOR CONTRIBUTIONS

**Fengyun Pei:** Data curation (equal); project administration (equal); writing – original draft (equal). **Wan He:** Data curation (equal); validation (equal); writing – original draft (equal). **Yinghua Duan:** Data curation (equal); project administration (equal); writing – original draft (equal). **Qijun Yao:** Data curation (equal); writing – original draft (equal). **Yandong Zhao:** Methodology (equal); resources (equal); supervision (equal). **Xinjuan Fan:** Formal analysis (equal); resources (equal). **Shuai Liu:** Data curation (equal); methodology (equal). **Haiyang Chen:** Data curation (equal); methodology (equal). **Fang He:** Data curation (equal); methodology (equal). **Tingzhi Liu:** Data curation (equal). **Jiaoting Chen:** Data curation (equal). **Yijia Zheng:** Data curation (equal). **Heping Li:** Data curation (equal). **Xiaofang Guo:** Data curation (equal). **Lishuo Shi:** Formal analysis (equal). **Li Ling:** Formal analysis (equal). **Yaoxu Chen:** Software (equal). **Jiapeng He:** Software (equal). **Miao Liu:** Software (equal). **Mengli Huang:** Software (equal). **Yuezong Bai:** Software (equal). **Jianping Wang:** Conceptualization (equal); project administration (equal). **Meijin Huang:** Project administration (equal); supervision (equal); writing – review and editing (equal). **Jun Huang:** Conceptualization (equal); funding acquisition (lead); investigation (equal); project administration (equal); writing – review and editing (equal).

## FUNDING INFORMATION

This study was supported by the National Key Clinical Discipline, the National Natural Science Foundation of China [Grant no. 81972885] and the 1010 Project of the Sixth Affiliated Hospital of Sun Yat‐sen University [1010CG (2020)‐20].

## CONFLICT OF INTEREST STATEMENT

Y.X.C., J.P.H., M.L., M.L.H. and Y.Z.B. were employed by Medical Affairs, 3D Medicines, Inc. The remaining authors declare that the research was conducted in the absence of any commercial or financial relationships that could be construed as a potential conflict of interest. The abstract of this study was selected for online publication at the 2022 ASCO Annual Meeting (submission ID: 362610, abstract number for publication: e15606).

## ETHICS STATEMENT

All procedures performed in this study involving human participants were in accordance with the ethical standards of the institutional and/or national research committee and with the 1964 Helsinki Declaration and its later amendments or comparable ethical standards. This study has received the approval (2022ZSLYEC‐43) of the ethics committee of the sixth Affiliated Hospital, Sun Yat‐sen University.

## Supporting information


**Data S1:** Supporting Information.

## Data Availability

The data used and/or analyzed during the current study are available from the corresponding author upon reasonable request.

## References

[1] Liu ZH , Wang N , Wang FQ , Dong Q , Ding J . Oncological outcomes of laparoscopic versus open surgery in pT4 colon cancers: a systematic review and meta‐analysis. Int J Surg. 2018;56:221‐233. 29940259 10.1016/j.ijsu.2018.06.032

[2] de Nes LCF , van der Heijden JAG , Verstegen MG , et al. Predictors of undergoing multivisceral resection, margin status and survival in Dutch patients with locally advanced colorectal cancer. Eur J Surg Oncol. 2022;48(5):1144‐1152. 34810058 10.1016/j.ejso.2021.11.004

[3] Leon P , Iovino MG , Giudici F , et al. Oncologic outcomes following laparoscopic colon cancer resection for T4 lesions: a case‐control analysis of 7‐years' experience. Surg Endosc. 2018;32(3):1133‐1140. 28842796 10.1007/s00464-017-5784-6

[4] Fang L , Yang Z , Zhang M , Meng M , Feng J , Chen C . Clinical characteristics and survival analysis of colorectal cancer in China: a retrospective cohort study with 13,328 patients from southern China. Gastroenterol Rep (Oxf). 2021;9(6):571‐582. 34925854 10.1093/gastro/goab048 PMC8677537

[5] Foxtrot Collaborative Group . Feasibility of preoperative chemotherapy for locally advanced, operable colon cancer: the pilot phase of a randomised controlled trial. Lancet Oncol. 2012;13(11):1152‐1160. 23017669 10.1016/S1470-2045(12)70348-0 PMC3488188

[6] Jung F , Guidolin K , Lee MH , et al. Interventions and outcomes for neoadjuvant treatment of T4 colon cancer: a scoping review. Curr Oncol. 2021;28(3):2065‐2078. 34072615 10.3390/curroncol28030191 PMC8261638

[7] Qiu B , Ding PR , Cai L , et al. Outcomes of preoperative chemoradiotherapy followed by surgery in patients with unresectable locally advanced sigmoid colon cancer. Chin J Cancer. 2016;35(1):65. 27389519 10.1186/s40880-016-0126-y PMC4936166

[8] Blank CU , Haanen JB , Ribas A , Schumacher TN . CANCER IMMUNOLOGY. The "cancer immunogram". Science. 2016;352(6286):658‐660. 27151852 10.1126/science.aaf2834

[9] Avallone A , Delrio P , Nasti G , et al. Preoperative nivolumab in patients (pts) with locally advanced colon cancer (T3 or T4): a window‐of‐opportunity study (NICOLE). Ann Oncol. 2018;29:viii203.

[10] Chalabi M , Fanchi LF , Dijkstra KK , et al. Neoadjuvant immunotherapy leads to pathological responses in MMR‐proficient and MMR‐deficient early‐stage colon cancers. Nat Med. 2020;26(4):566‐576. 32251400 10.1038/s41591-020-0805-8

[11] Wallin JJ , Bendell JC , Funke R , et al. Atezolizumab in combination with bevacizumab enhances antigen‐specific T‐cell migration in metastatic renal cell carcinoma. Nat Commun. 2016;7:12624. 27571927 10.1038/ncomms12624 PMC5013615

[12] Le DT , Uram JN , Wang H , et al. PD‐1 blockade in tumors with mismatch‐repair deficiency. N Engl J Med. 2015;372(26):2509‐2520. 26028255 10.1056/NEJMoa1500596 PMC4481136

[13] Overman MJ , McDermott R , Leach JL , et al. Nivolumab in patients with metastatic DNA mismatch repair‐deficient or microsatellite instability‐high colorectal cancer (CheckMate 142): an open‐label, multicentre, phase 2 study. Lancet Oncol. 2017;18(9):1182‐1191. 28734759 10.1016/S1470-2045(17)30422-9 PMC6207072

[14] Antoniotti C , Rossini D , Pietrantonio F , et al. Upfront FOLFOXIRI plus bevacizumab with or without atezolizumab in the treatment of patients with metastatic colorectal cancer (AtezoTRIBE): a multicentre, open‐label, randomised, controlled, phase 2 trial. Lancet Oncol. 2022;23(7):876‐887. 35636444 10.1016/S1470-2045(22)00274-1

[15] Fang X , Zhu N , Zhong C , et al. Sintilimab plus bevacizumab, oxaliplatin and capecitabine as first‐line therapy in RAS‐mutant, microsatellite stable, unresectable metastatic colorectal cancer: an open‐label, single‐arm, phase II trial. EClinicalMedicine. 2023;62:102123. 37554125 10.1016/j.eclinm.2023.102123 PMC10404864

[16] Hu H , Huang M , Li Y , et al. Perioperative chemotherapy with mFOLFOX6 or CAPOX for patients with locally advanced colon cancer (OPTICAL): a multicenter, randomized, phase 3 trial. https://meetings.asco.org/abstracts‐presentations/208328

[17] Takano A , Daigo Y . Assessment of anticancer drug‐induced adverse event. Nihon Rinsho. 2015;73(Suppl. 2):75‐78. 25831727

[18] Freites‐Martinez A , Santana N , Arias‐Santiago S , Viera A . Using the common terminology criteria for adverse events (CTCAE—version 5.0) to evaluate the severity of adverse events of anticancer therapies. Actas Dermosifiliogr (Engl Ed). 2021;112(1):90‐92. 32891586 10.1016/j.ad.2019.05.009

[19] Eisenhauer EA , Therasse P , Bogaerts J , et al. New response evaluation criteria in solid tumours: revised RECIST guideline (version 1.1). Eur J Cancer. 2009;45(2):228‐247. 19097774 10.1016/j.ejca.2008.10.026

[20] Merlo LM , Pepper JW , Reid BJ , Maley CC . Cancer as an evolutionary and ecological process. Nat Rev Cancer. 2006;6(12):924‐935. 17109012 10.1038/nrc2013

[21] Brierley JD , Gospodarowicz MK , Wittekind C . Skin tumours. TNM Classification of Malignant Tumours. John Wiley & Sons; 2017.

[22] Liang Y , Zhu Y , Lin H , et al. The value of the tumour‐stroma ratio for predicting neoadjuvant chemoradiotherapy response in locally advanced rectal cancer: a case control study. BMC Cancer. 2021;21(1):729. 34172021 10.1186/s12885-021-08516-x PMC8235870

[23] Lenz H‐J , Parikh AR , Spigel DR , et al. Nivolumab (NIVO) + 5‐fluorouracil/leucovorin/oxaliplatin (mFOLFOX6)/bevacizumab (BEV) versus mFOLFOX6/BEV for first‐line (1L) treatment of metastatic colorectal cancer (mCRC): phase 2 results from CheckMate 9X8. J Clin Oncol. 2022;40(4_suppl):8.34694897

[24] Hu H , Kang L , Zhang J , et al. Neoadjuvant PD‐1 blockade with toripalimab, with or without celecoxib, in mismatch repair‐deficient or microsatellite instability‐high, locally advanced, colorectal cancer (PICC): a single‐centre, parallel‐group, non‐comparative, randomised, phase 2 trial. Lancet Gastroenterol Hepatol. 2022;7(1):38‐48. 34688374 10.1016/S2468-1253(21)00348-4

[25] Lee AH , Sun L , Mochizuki AY , et al. Neoadjuvant PD‐1 blockade induces T cell and cDC1 activation but fails to overcome the immunosuppressive tumor associated macrophages in recurrent glioblastoma. Nat Commun. 2021;12(1):1‐16. 34836966 10.1038/s41467-021-26940-2 PMC8626557

[26] Fukumura D , Kloepper J , Amoozgar Z , Duda DG , Jain RK . Enhancing cancer immunotherapy using antiangiogenics: opportunities and challenges. Nat Rev Clin Oncol. 2018;15(5):325‐340. 29508855 10.1038/nrclinonc.2018.29 PMC5921900

[27] Taniura T , Iida Y , Kotani H , Ishitobi K , Tajima Y , Harada M . Immunogenic chemotherapy in two mouse colon cancer models. Cancer Sci. 2020;111(10):3527‐3539. 32816355 10.1111/cas.14624 PMC7541014

[28] Bartlett EK , Flynn JR , Panageas KS , et al. High neutrophil‐to‐lymphocyte ratio (NLR) is associated with treatment failure and death in patients who have melanoma treated with PD‐1 inhibitor monotherapy. Cancer. 2020;126(1):76‐85. 31584709 10.1002/cncr.32506 PMC6906249

[29] Matsutani S , Shibutani M , Maeda K , et al. Significance of tumor‐infiltrating lymphocytes before and after neoadjuvant therapy for rectal cancer. Cancer Sci. 2018;109(4):966‐979. 29464828 10.1111/cas.13542 PMC5891199

[30] Jary M , Ww L , Yan D , et al. The immune microenvironment in patients with mismatch‐repair‐proficient oligometastatic colorectal cancer exposed to chemotherapy: the randomized MIROX GERCOR cohort study. Mol Oncol. 2022;16(11):2260‐2273. 34954864 10.1002/1878-0261.13173 PMC9168761

[31] Mlecnik B , Tosolini M , Kirilovsky A , et al. Histopathologic‐based prognostic factors of colorectal cancers are associated with the state of the local immune reaction. J Clin Oncol. 2011;29(6):610‐618. 21245428 10.1200/JCO.2010.30.5425

[32] Liu H , Zhang Z , Zhen P , Zhou M . High expression of VSTM2L induced resistance to chemoradiotherapy in rectal cancer through downstream IL‐4 signaling. J Immunol Res. 2021;2021:1‐17. 10.1155/2021/6657012 PMC7811563 33506057

