# Supplementary material for: PD‐1 blockade enhances the effect of targeted chemotherapy on locally advanced pMMR/MSS colorectal cancer
Source: Cancer Med. 2024 Jun 18;13(12):e7224. doi: 10.1002/cam4.7224 (PMC11184646; doi:10.1002/cam4.7224)
Supplement: Supplementary file 1 — Data S1: Supporting Information. [file CAM4-13-e7224-s001.docx]

**SUPPLEMENTARY TABLES**

**Supplementary Table 1. Pretreatment clinical stage and posttreatment pathological stage**

| Patient number | Pretreatment  clinical stage,  TNM (stage group) | Preoperative  clinical stage  TNM, (stage group) | Pathological stage at resection,  TNM (stage group) | | Radiological downstaging (yes/no) | Pathological  downstaging  (yes/no) | |
| --- | --- | --- | --- | --- | --- | --- | --- |
| 1 | T4bN2 (IIIC) | T4bN1 (IIIC) | T0N0 (0) | No | | Yes | |
| 2 | T4bN2 (IIIC) | T4bN1 (IIIC) | T0N0 (0) | No | | Yes | |
| 3 | T4bN2 (IIIC) | T3N0 (IIA) | T3N0 (IIA) | Yes | | Yes |  |
| 4 | T4bN2 (IIIC) | T3N1 (IIIB) | T0N0 (0) | Yes | | Yes | |
| 5 | T4aN1 (IIIB) | T3-4aN0 (IIA-B) | T0N0 (0) | Yes | | Yes | |
| 6 | T4aN1 (IIIB) | T4aN1 (IIIB) | T0N0 (0) | No | | Yes | |
| 7 | T4aN2 (IIIC) | T3N1 (IIIB) | T0N0 (0) | Yes | | Yes | |
| 8 | T4aN2 (IIIC) | T3N1 (IIIB) | T2N0 (I) | Yes | | Yes | |
| 9 | T4aN1 (IIIB) | T3-4aN0 (IIA-B) | T3N0 (IIA) | Yes | | Yes | |
| 10 | T4aN1 (IIIB) | T4aN1 (IIIB) | T3N2a (IIIB) | No | | No | |
| 11 | T4aN2 (IIIC) | T3N2 (IIIB-C) | T1N0 (I) | Yes | | Yes | |
| 12 | T4aN2 (IIIC) | T2N1 (IIIA) | T2N1 (IIIA) | Yes | | Yes | |
| 13 | T4aN2 (IIIC) | T3bN1 (IIIB) | T0N0 (0) | Yes | | Yes | |
| 14 | T4bN2 (IIIC) | T3N0 (IIA) | T0N0 (0) | Yes | | Yes | |
| 15 | T4bN2 (IIIC) | T4aN0 (IIB) | T0N0 (0) | Yes | | Yes | |
| 16 | T4bN2 (IIIC) | T4aN1 (IIIB) | T0N0 (0) | Yes | | Yes | |
| 17 | T4bN2 (IIIC) | T4aN2 (IIIC) | T4aN2b (IIIC) | No | | No | |
| 18 | T4aN2 (IIIC) | T4aN0 (IIB) | T3N0 (IIA) | Yes | | Yes | |
| 19 | T4aN2 (IIIC) | T3N1 (IIIB) | T1N0 (I) | Yes | | Yes | |
| 20 | T4aN1 (IIIB) | T4aN1 (IIIB) | T0N0 (0) | No | | Yes | |
| 21 | T4bN2 (IIIC) | T4bN2 (IIIC) | T1N0 (I) | No | | Yes | |
| 22 | T4aN2 (IIIC) | T3bN1 (IIIB) | T0N0 (0) | Yes | | Yes | |

**Supplementary Table 2. Treatment-related adverse events.**

| Event (N=22) | Any (%) | Grade≥3 (%) |
| --- | --- | --- |
| Decreased white blood cell count | 7 (32) | 1 (5) |
| Neutropenia | 14 (64) | 3 (14) |
| Anemia | 10 (45) | 1 (5) |
| Thrombocytopenia | 2 (9) | 0 |
| Increase in alanine aminotransferase | 2 (9) | 1 (5) |
| Increase in aspartate aminotransferase | 2 (9) | 1 (5) |
| Decreased appetite | 11 (50) | 0 |
| Nausea | 5 (23) | 0 |
| Vomiting | 6 (27) | 0 |
| Abdominal pain | 3 (14) | 0 |
| Fatigue | 7 (32) | 0 |
| Diarrhea | 4 (18) | 0 |
| Peripheral neuropathy | 9 (41) | 0 |
| Dysgeusia | 5 (23) | 0 |
| Dry skin | 2 (9) | 0 |
| Rash | 1 (5) | 0 |
| Pruritus | 1 (5) | 0 |
| Pneumonia | 0 | 0 |
| Dizziness | 6 (27) | 0 |
| Infusion reaction | 0 | 0 |
| Hypothyroidism | 1 (5) | 0 |
| Arthralgia | 3 (14) | 0 |
| Pyrexia | 2 (9) | 0 |
| Myalgia | 1 (5) | 0 |
| Elevated creatinine | 1 (5) | 0 |
| Hypertension | 2 (9) | 0 |
| Oral mucositis | 2 (9) | 0 |
| Dry mouth | 4 (18) | 0 |
| Chest tightness | 1 (5) | 0 |

**Supplementary Figure 1. Study design and correlation analyses**

**
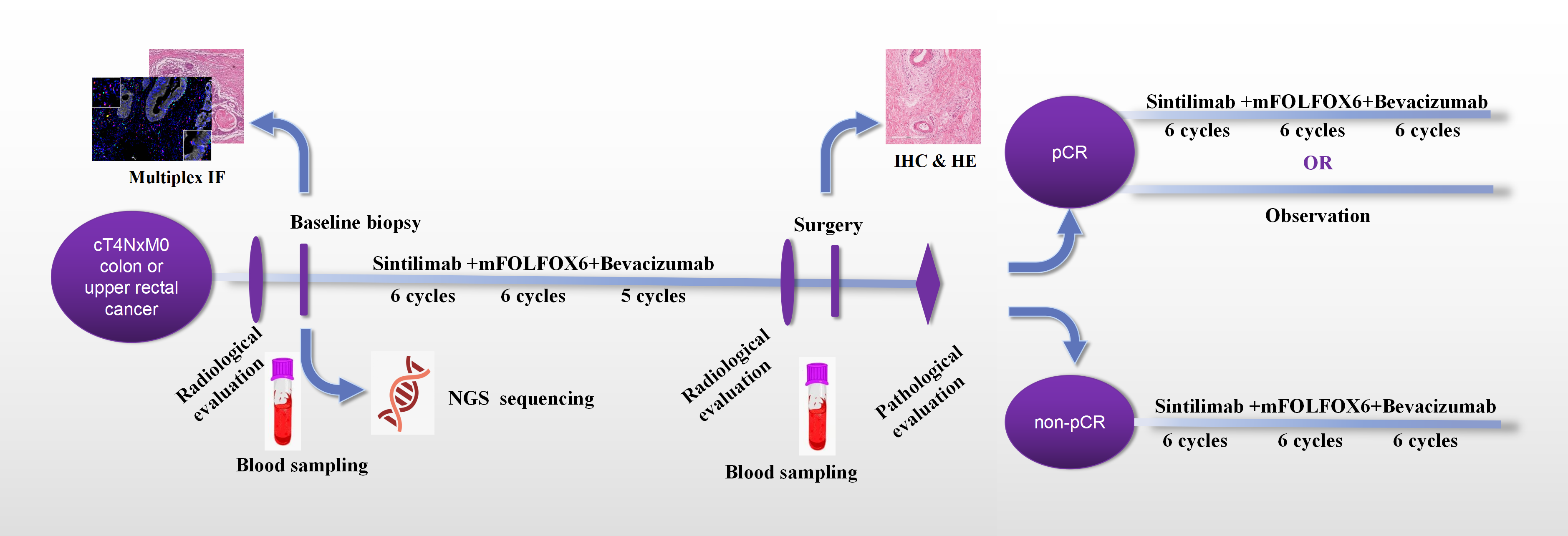
**

Sintilimab (at a fixed dose of 200 mg) in combination with mFOLFOX6 for 6 cycles and 5 mg/kg^2^ bevacizumab for 5 cycles was administered every two weeks prior to surgical resection. Tumor samples were collected at baseline and at the time of resection, and serial blood samples were collected at baseline and at the time of resection. Multiplex immunofluorescence (IF) was performed with selected pretreatment tumor and tumor resection specimens. In cases where sufficient tissues were available, immunohistochemistry assays were performed as follows. Next-generation sequencing was conducted with matched pretreatment tumor and stromal samples.

**Supplementary Figure 2. Differences in Immune Cells Before and After Neoadjuvant Therapy with a PD-1 Antibody in Combination with mFOLFOX6 and Bevacizumab.**

**B**  **Postoperative Pretreatment**

**treatmentPretreatment**

**A Postoperative Pretreatment**

**
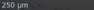

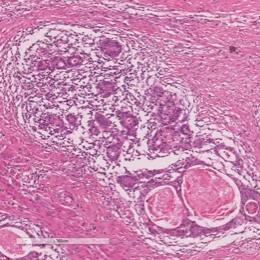

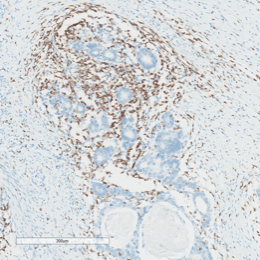

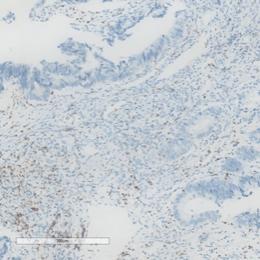

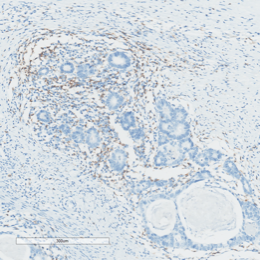

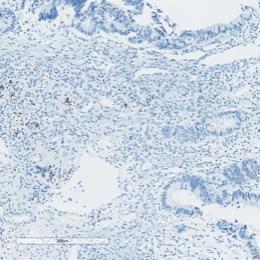

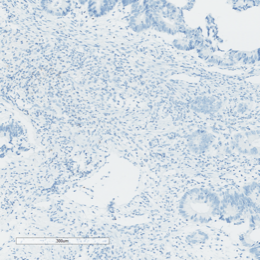

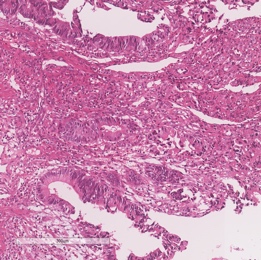

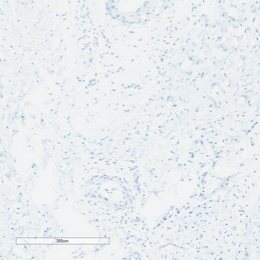

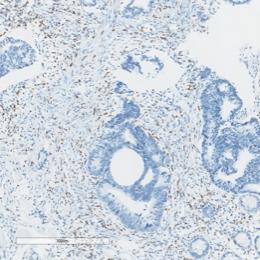

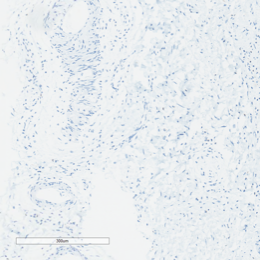

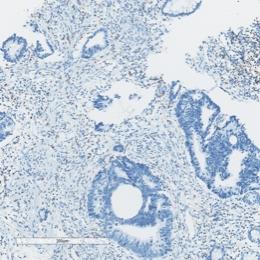

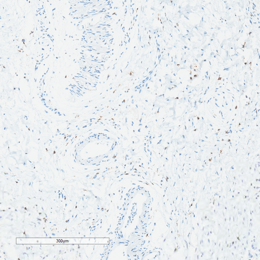

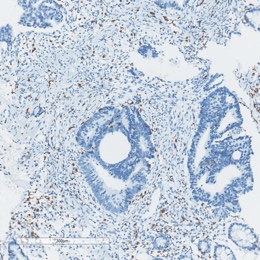

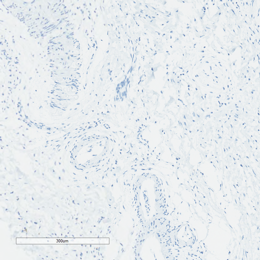

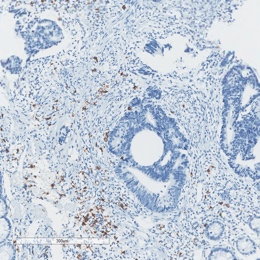

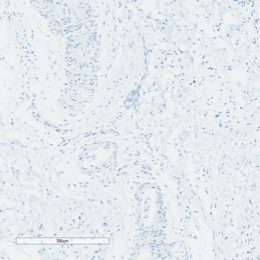

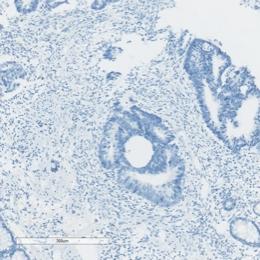

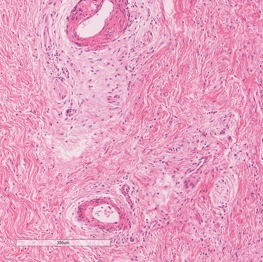

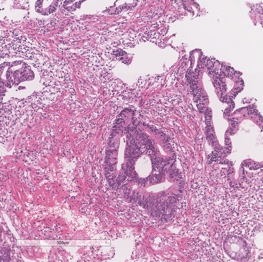

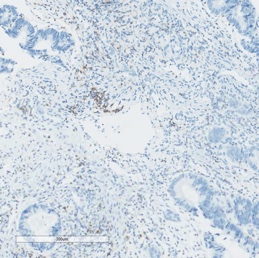

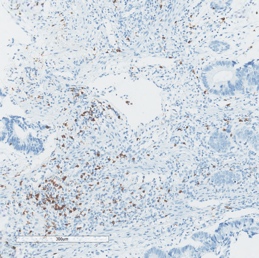

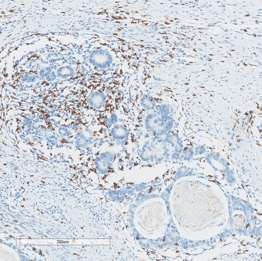

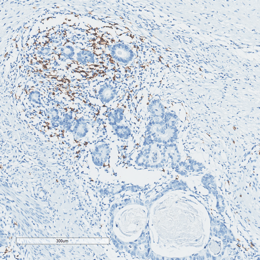

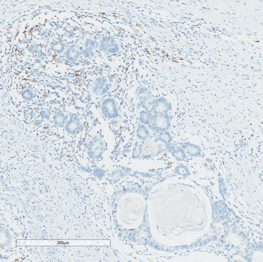
**

HE

CD3

CD4

CD8

CD20

PD-1

**
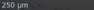

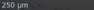
**

**
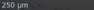
**

**
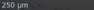

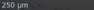

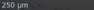

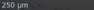
**

**
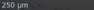

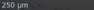

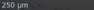

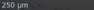
**

**
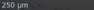

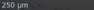

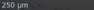

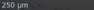
**

**
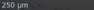

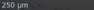

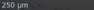

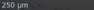
**

**
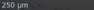

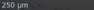

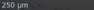

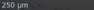
**

The expression of CD3, CD4, CD8, CD20 and PD-1 are shown by immunohistochemical staining in pretreatment and postoperative specimens. Panel A shows patients who received neoadjuvant treatment and achieved nonpCR (left). All the above antigen-antibody staining images in postoperative specimens show scattered tumor cell nests. Panel B shows patients who received neoadjuvant treatment and achieved pCR (right); all above antigen-antibody staining images from the postoperative specimens are negative.

**Supplementary Figure 3. Correlation of Pathological and Radiological Tumor Regression after Neoadjuvant Treatment.**


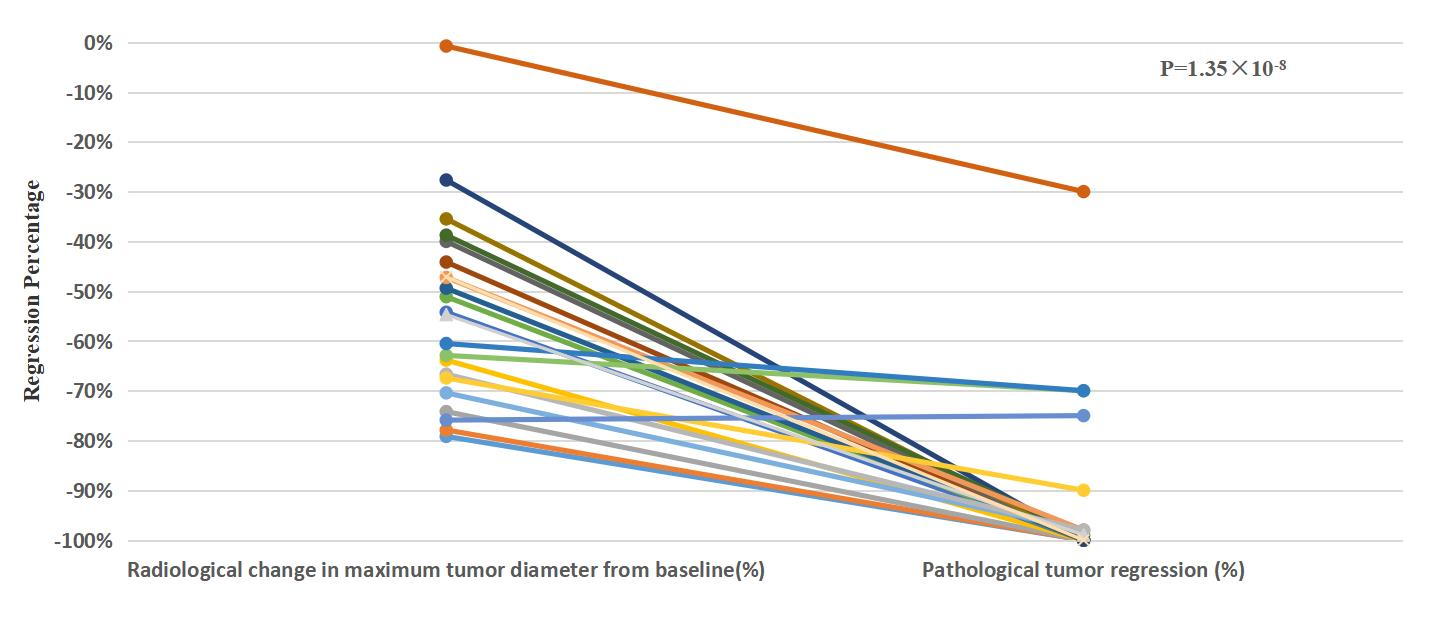


The percentages of radiological change in maximum tumor diameter from baseline (%) and pathological tumor regression (%) are shown. Each color represents an individual patient. A t test was performed to explore the difference between the two groups. A P value less than 0.05 indicated a significant difference.

**Supplementary Figure 4. Recurrence-free Survival (RFS) in pMMR/MSS Locally Advanced CRC Patients.**


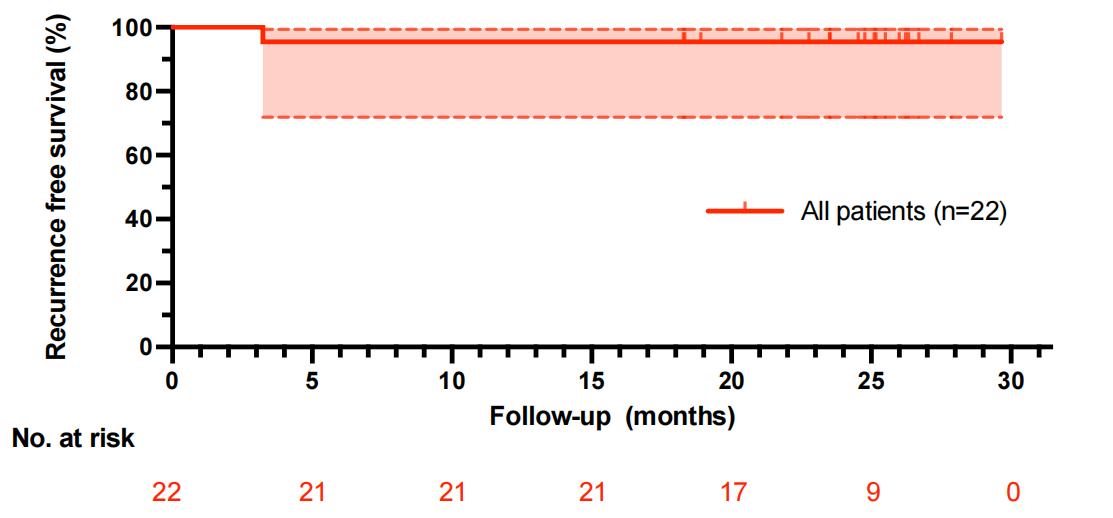


Kaplan–Meier RFS estimates with 95% confidence intervals are shown. RFS was calculated from the time of surgery to the time of recurrence or death. The median follow-up was 24.7 months. The numbers of patients at risk in 5-month intervals are included below the x-axis.

**Supplementary Figure 5. Landscape of Genomic Mutations.**


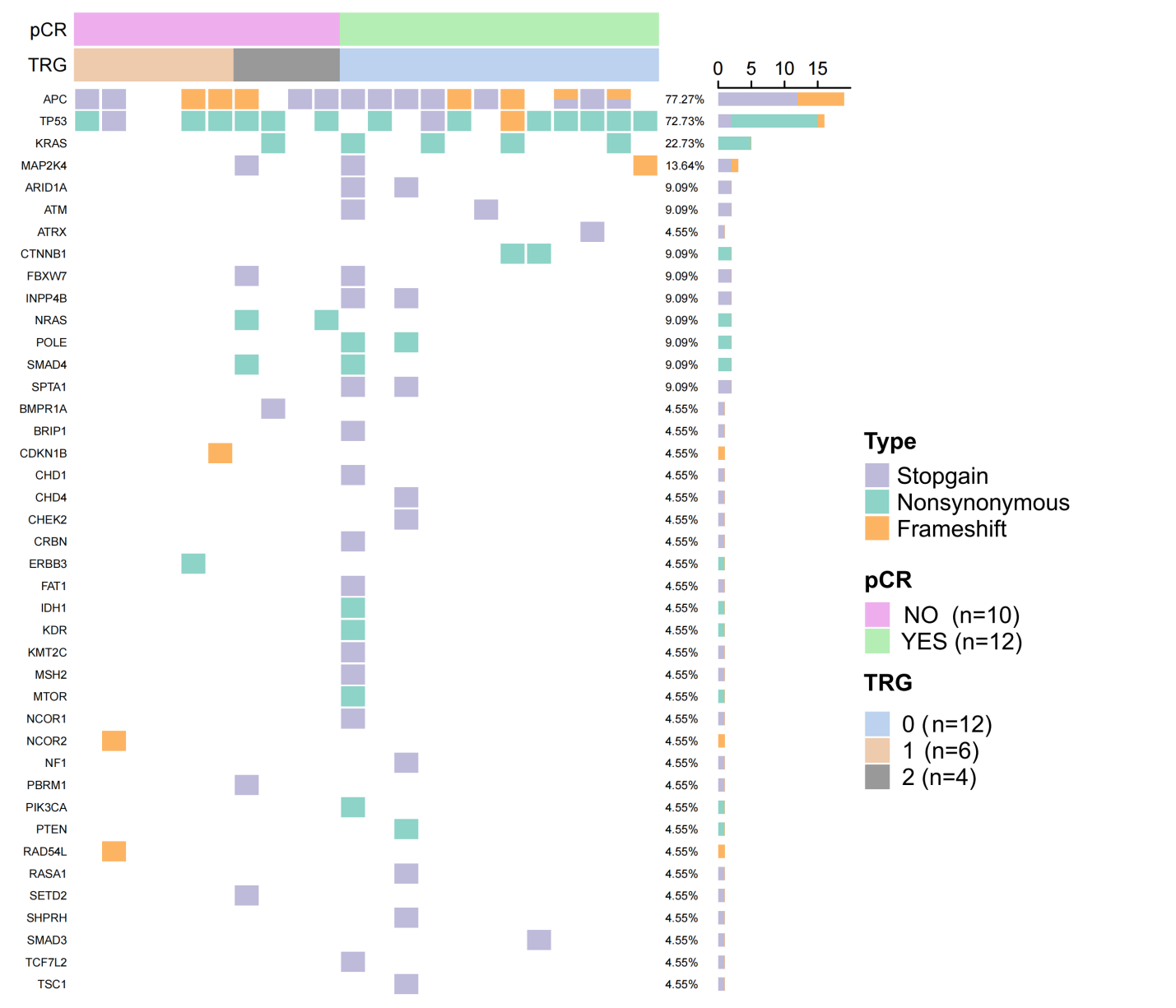


The mutation patterns of selected genes, including stop-gain, nonsynonymous and frameshift mutations, according to patients’ pCR status (yes or no) and TRG (0-2). The mutation rate of each gene is also shown.

**Supplementary Figure 6. Association between Tumor Mutation Burden and the Pathologic Response to Neoadjuvant Therapy with a PD-1 Antibody in Combination with mFOLFOX6 and Bevacizumab**

**A.**

**
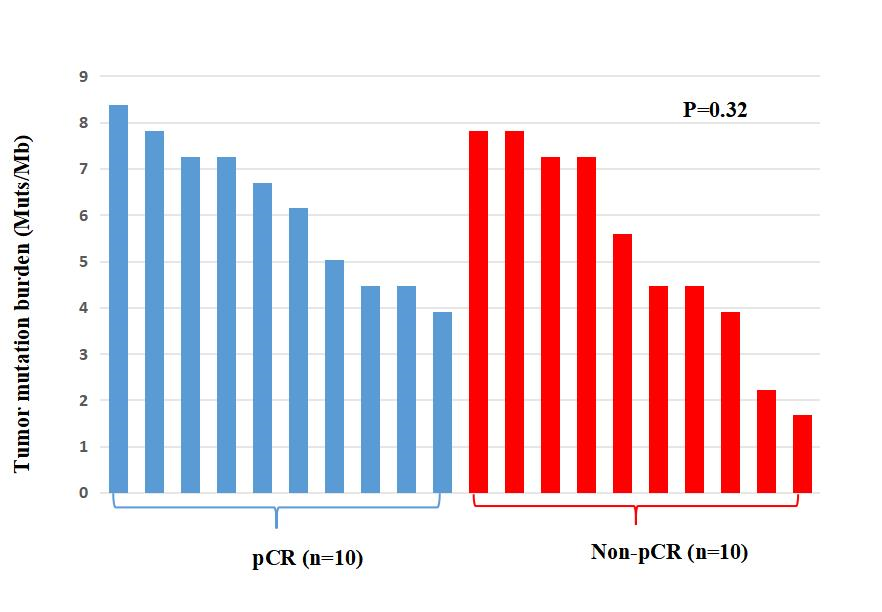
**

**B.**

**
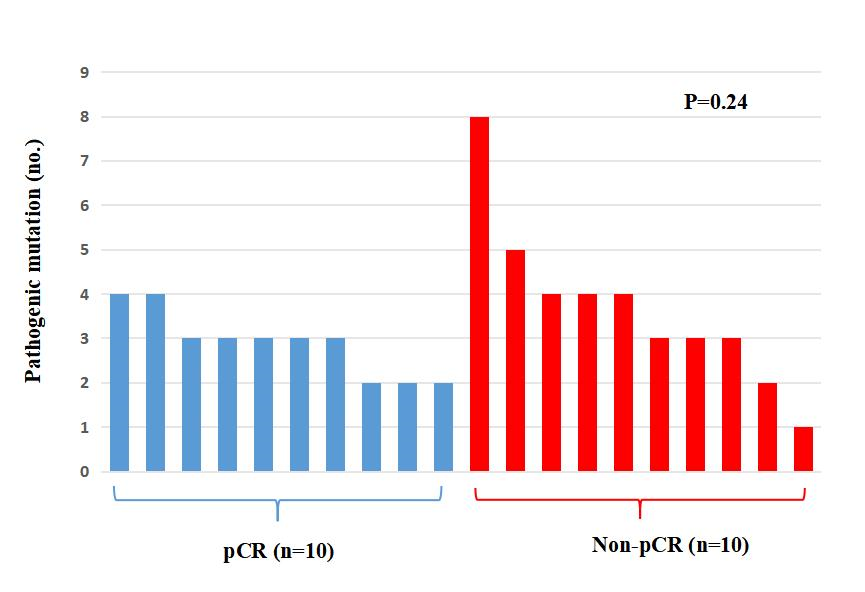
**

1. The tumor mutation burden (TMB) in pretreatment tumor samples obtained from 20 patients who underwent surgical resection and had sufficient pretreatment tissue available for sequencing, exhibiting differential responses to PD-1 blockade. Patients without POLE mutations who had a pathologic complete response were found to have a tendency toward higher TMB than those without a pathologic complete response, with a mean (±SE) TMB of 6.15±1.58 and 5.25±2.27 (P = 0.32; exact Wilcoxon test).
2. The number of pathogenic mutations in pretreatment tumor samples obtained from 20 patients who underwent surgical resection. Patients without POLE mutations who had a pathologic complete response were found to have a lower number of pathogenic mutation tendencies than those without a pathologic complete response, with a mean (±SE) of 2.90±0.74 and 3.70±1.89 mutations (P = 0.24; exact Wilcoxon test).

**Supplementary Figure 7. Correlation between the Number of Sequence Alterations and Percentage of Residual Tumor.**

**A**

**
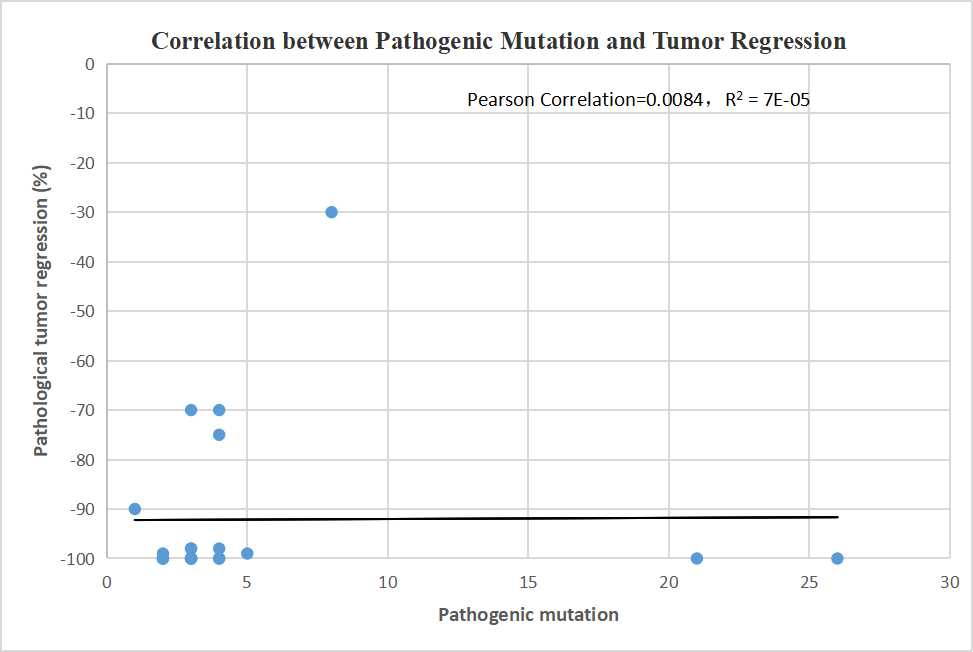
**

**B**

**
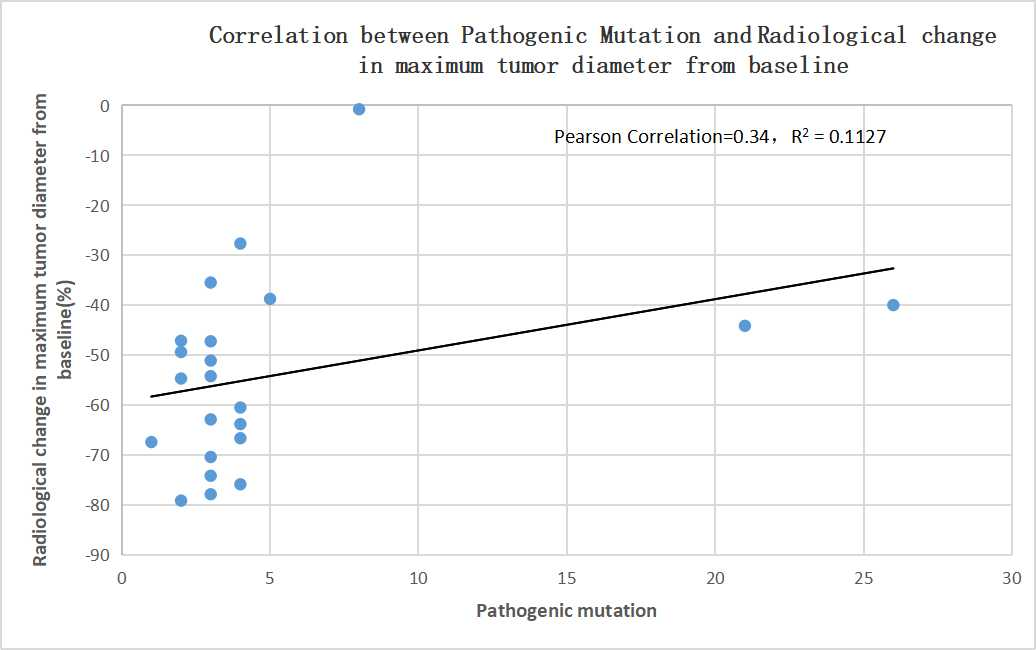
**

1. The correlation between pathogenic mutation number and pathological tumor regression (n=22). The number of pathogenic mutations was correlated with the pathological tumor regression (Pearson correlation=0.0084, R^2^=7×10^-5^).
2. The correlation between pathogenic mutation number and radiological tumor regression (n=22). The number of pathogenic mutations was not correlated with radiological tumor regression (Pearson correlation=0.34, R^2^=0.1127).

**Supplementary Figure 8. Correlation of TME Characteristics in the Biopsy Before Neoadjuvant Treatment and Tumor Regression Grade.**

The correlation between the tumor microenvironment (TME) characteristics and tumor regression grade (TRG) is shown. (A) TME characteristics among different TRGs. Patients with TRG1 were found to have a higher CD3+CD4+ percentage than those with TRG0 and TRG2 in the stromal region (p=0.020; Kruskal–Wallis test). Representative pictures of pCR patient No. 14 (B) and nonpCR patient No. 2 (C). Multiplex immunofluorescence staining was conducted using the Akoya OPAL Polaris 7-Color Automation IHC kit (NEL871001KT). FFPE tissue slides were incubated with primary antibodies targeting CD163 (red color, Abcam, ab182422, 1:500), CD68 (cyan color, Abcam, ab213363, 1:1000), PD-1 (green color, CST, D4W2J, 86163S, 1:200), PD-L1 (yellow color, CST, E1L3N, 13684S, 1:400), CD8 (pink color, Abcam, ab178089, 1:100) and pan-CK (white color, Abcam, ab7753, 1:100), which allowed the identification of different immune cell subtypes (CD8+ T cells, M1 macrophages, M2 macrophages, PD-1 expression and PD-L1 expression).

**Supplementary Figure 9.** **Correlation between NLR (at Diagnosis and before Surgery) and Percentage of Residual Tumor.**

**A**

**
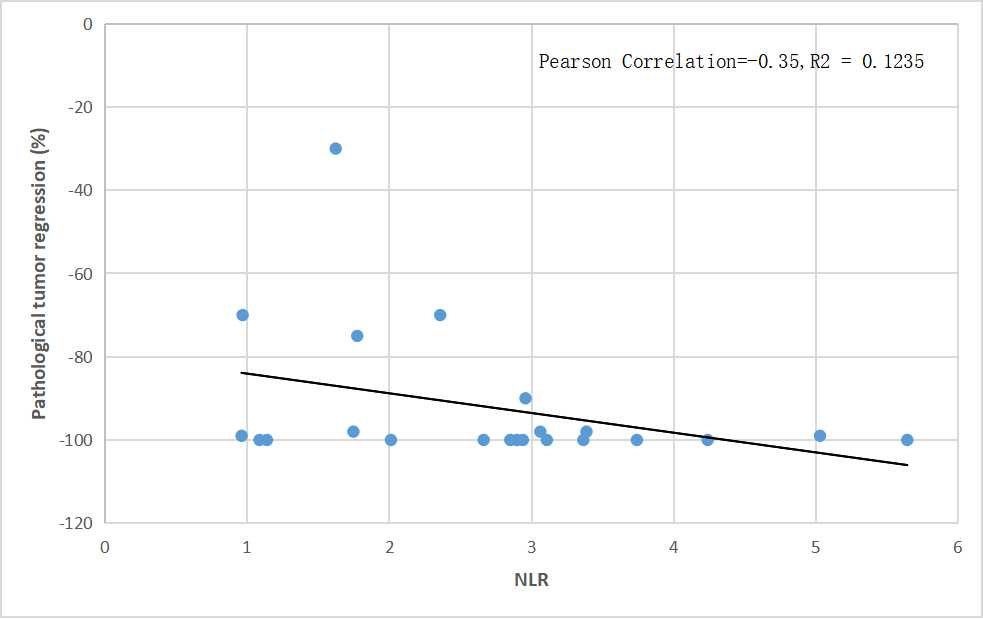
**

**B**

**
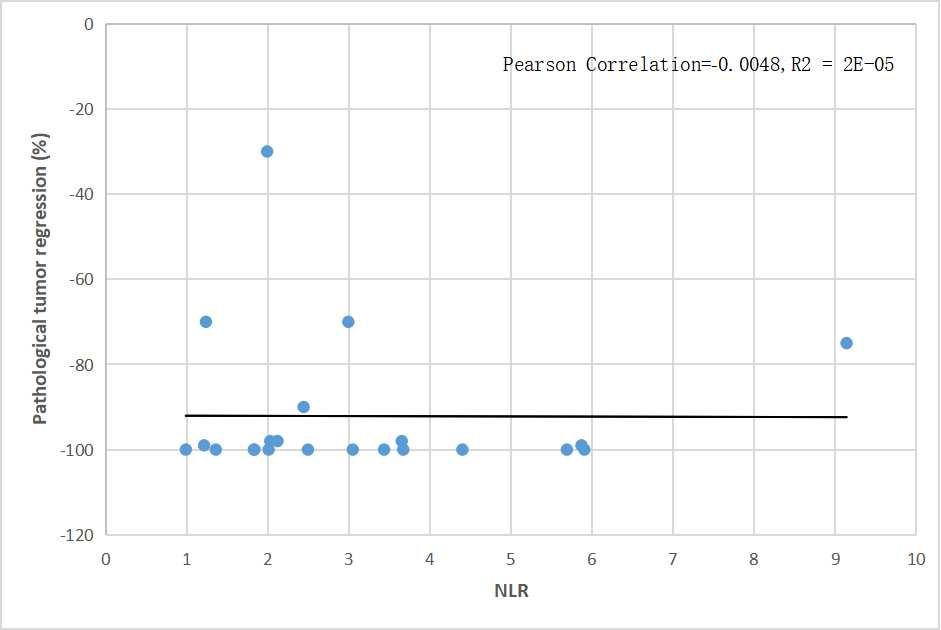
**

A. Correlation between the neutrophil-to-lymphocyte ratio (NLR) at diagnosis and pathological tumor regression (n=22). The NLR was not correlated with pathological tumor regression (Pearson correlation=0.35, R^2^=0.1235).

B. Correlation between NLR before surgery and radiological tumor regression (n=22). The NLR was correlated with pathological tumor regression (Pearson correlation=0.0048, R^2^=2×10^-5^).

**SUPPLEMENTARY METHODS**

**Pathology**

**Immunohistochemical analysis**

The entire tumor tissue was submitted for microscopic examination. Formalin-fixed, paraffin-embedded (FFPE) tissue samples were obtained. The 4-μm sections were stained with hematoxylin and eosin (H&E) and examined. Immunohistochemical analysis was performed using the BenchMark XT Automatic Immunohistochemical Staining Instrument (Roche, Switzerland). Commercially available monoclonal antibodies against CD3 (ZSGB-BIO, China), CD4 (ZSGB-BIO, China), CD8 (ZSGB-BIO, China), CD20 (Roche, Switzerland), and PD1 (ZSGB-BIO, China) were used according to established protocols.

| Position | Antibody | Clone (host)/Company | Dilution | Incubation | TSA Dyes |
| --- | --- | --- | --- | --- | --- |
| 1 | PD-1 | ZM-0381/ZSGB-BIO | No dilution | 37 ℃ for 32 min | - |
| 2 | CD3 | ZA-0503/ZSGB-BIO | No dilution | 37 ℃ for 40 min | - |
| 3 | CD4 | ZA-0519/ZSGB-BIO | No dilution | 37 ℃ for 28 min | - |
| 4 | CD8 | ZA-0508/ZSGB-BIO | No dilution | 37 ℃ for 16 min | - |
| 5 | CD20 | H04556/Roche | No dilution | 37 ℃ for 16 min | - |

**Tissue processing and genomic DNA extraction**

FFPE tissue sections were evaluated for tumor cell content using H&E staining. Only samples with a tumor content ≥20% were eligible for subsequent analyses. FFPE tissue sections were placed in a 1.5 microcentrifuge tube and deparaffinized with mineral oil. Samples were incubated with lysis buﬀer and proteinase K at 56 °C overnight until the tissue was completely digested. The lysate was subsequently incubated at 80 °C for 4 hours to reverse formaldehyde crosslinks. Genomic DNA was isolated from tissue samples using the ReliaPrep™ FFPE gDNA Miniprep System (Promega) and quantified using the Qubit™ dsDNA HS Assay Kit (Thermo Fisher Scientific) following the manufacturer’s instructions.

**Library preparation and targeted capture**

DNA extracts (30-200 ng) were sheared to 250 bp fragments using an S220 focused ultrasonicator (Covaris). Libraries were prepared using the KAPA Hyper Prep Kit (KAPA Biosystems) following the manufacturer’s protocol. The concentration and size distribution of each library were determined using a Qubit 3.0 fluorometer (Thermo Fisher Scientific) and a LabChip GX Touch HT Analyzer (PerkinElmer), respectively.

For targeted capture, indexed libraries were subjected to probe-based hybridization with a customized NGS panel targeting 733 cancer-related genes, where the probe baits were individually synthesized 5′-biotinylated 120-bp DNA oligonucleotides (IDT). Repeated elements were filtered out from intronic baits according to UCSC Genome RepeatMasker annotation. ^1^ The xGen® Hybridization and Wash Kit (IDT) was employed to enrich hybridization. Briefly, 500 ng of indexed DNA libraries were pooled to obtain a total amount of 2 μg of DNA. The pooled DNA sample was then mixed with human cot DNA and xGen Universal Blockers-TS Mix and dried in a SpeedVac system. Hybridization Master Mix was added to the samples and they were incubated in a thermal cycler at 95 °C for 10 min before being mixed and incubated with 4 μl of probes at 65 °C overnight. The target regions were captured following the manufacturer’s instructions. The concentration and fragment size distribution of the final library were determined using a Qubit 3.0 fluorometer (Thermo Fisher Scientific) and a LabChip GX Touch HT Analyzer (PerkinElmer), respectively.

**DNA sequencing, data processing, and variant calling**

The captured libraries were loaded onto a NovaSeq 6000 platform (Illumina) for 100-bp paired-end sequencing with a mean sequencing depth of 500X.

Raw data from paired samples (an FFPE sample and its normal tissue control) were mapped to the reference human genome hg19 using the Burrows–Wheeler Aligner (v0.7.12). ^2^ PCR duplicate reads were removed, and sequence metrics were collected using Picard (v1.130) and SAMtools (v1.1.19). Variant calling was performed only in the targeted regions. Somatic single nucleotide variants (SNVs) were detected using an in-house R package that executed a variant detection model based on a binomial test. Local realignment was performed to detect indels. Variants were then filtered by their unique supporting read depth, strand bias, and base quality as previously described.^3^ All variants were then filtered using an automated false positive filtering pipeline to ensure sensitivity and specificity at an allele frequency (AF) of ≥ 1%. Single-nucleotide polymorphisms (SNPs) and indels were annotated by ANNOVAR against the following databases: dbSNP (v138), 1000Genome and ESP6500 (population frequency > 0.015). Only missense, stop-gain, frameshift and nonframeshift indel mutations were retained. Copy number variations (CNVs) and gene rearrangements were detected as described previously.^3^

TMB was defined as the number of synonymous and nonsynonymous somatic SNVs and indels in examined coding regions, with driver mutations excluded. All SNVs and indels in the coding region of targeted genes, including missense, silent, stop-gain, stop-loss, frameshift and nonframeshift mutations, were considered.

**mIF investigation of the TME**

Multiplex immunofluorescence staining was conducted using the Akoya OPAL Polaris 7-Color Automation IHC kit (NEL871001KT). FFPE tissue slides were first deparaffinized in a BOND RX system (Leica Biosystems) and then incubated sequentially with primary antibodies targeting CD163 (Abcam, ab182422, 1:500), CD68 (Abcam, ab213363, 1:1000), PD-1 (CST, D4W2J, 86163S, 1:200), PD-L1 (CST, E1L3N, 13684S, 1:400), CD3 (Dako, A0452), CD4 (Abcam, ab133616, 1:100), CD8 (Abcam, ab178089, 1:100), CD56 (Abcam, ab75813, 1:100), CD20 (Dako, L26, IR604), FOXP3 (Abcam, ab20034, 1:100) and pan-CK (Abcam, ab7753, 1:100) (Akoya Biosciences). This incubation was followed by incubation with secondary antibodies and corresponding reactive Opal fluorophores. Nucleic acids were stained with DAPI. Tissue slides that were bound with primary and secondary antibodies but not fluorophores were included as negative controls to assess autofluorescence. Multiplex-stained slides were scanned using a Vectra Polaris Quantitative Pathology Imaging System (Akoya Biosciences) at 20-nm wavelength intervals from 440 nm to 780 nm with a fixed exposure time and an absolute magnification of 200×. All scans for each slide were then superimposed to obtain a single image. Multilayer images were imported into inForm v.2.4.8 (Akoya Biosciences) for quantitative image analysis. Tumor parenchyma and stroma were differentiated by Pan-CK staining using HALO software from Indica labs. The quantities of various cell populations were expressed as the number of stained cells per square millimeter and as the percentage of positively stained cells in all nucleated cells.

**References:**

1. Karolchik, D., et al., *The UCSC Table Browser data retrieval tool.* Nucleic Acids Res, 2004. **32**(Database issue): p. D493-6.

2. Li, H. and R. Durbin, Fast and accurate short read alignment with Burrows-Wheeler transform. Bioinformatics, 2009. 25(14): p. 1754-60.

3. Su, D., et al., High performance of targeted next generation sequencing on variance detection in clinical tumor specimens in comparison with current conventional methods. J Exp Clin Cancer Res, 2017. 36(1): p. 121.
